# Nanodefect-Mediated Strengthening and Deformation Mechanisms in Magnesium Alloys: A Critical Review

**DOI:** 10.3390/nano16110699

**Published:** 2026-06-05

**Authors:** Nürettin Akçakale, Muhammad Ishtiaq, Temel Varol, Mohsen Saboktakin Rizi

**Affiliations:** 1 Gerede Vocational School, Bolu Abant Izzet Baysal University, 14030 Bolu, Turkey; 2 Department of Materials Engineering and Convergence Technology, Gyeongsang National University, Jinju 52828, Republic of Korea; 3 Department of Metallurgical and Materials Engineering, Karadeniz Technical University, 61080 Trabzon, Turkey; tvarol@ktu.edu.tr; 4 Department of Mechanical Engineering, Incheon National University, Incheon 22012, Republic of Korea

**Keywords:** Mg alloys, nanoprecipitates, nano-stacking faults, nanotwins, mechanical properties

## Abstract

Nanodefect engineering has emerged as an effective strategy to address the inherent strength–ductility trade-off and limited damage tolerance of wrought and cast magnesium alloys through controlled manipulation of their defect structures. Recent advances demonstrate that introducing and tailoring nanoscale defects can significantly enhance mechanical performance and, under appropriate defect architectures and processing conditions, may enable improved strength–ductility balance. This review provides a concise, mechanism-oriented overview of nanodefect-mediated strengthening in Mg alloys, focusing on the roles of nanograins, nanoprecipitates, nanotwins, and nano-stacking faults. Grain refinement via severe plastic deformation and other processing routes enhances strength through Hall–Petch effects while modifying texture and activating non-basal slip. Concurrently, nanoscale precipitates contribute through dislocation shearing and Orowan bypassing, whereas planar defects such as nanotwins and stacking faults introduce high-density interfaces that both impede dislocation motion and facilitate plastic accommodation. Emphasis is placed on the synergistic interactions among these defect populations, which govern strain hardening, deformation stability, and the overall strength–ductility balance. The review underscores that tailored defect architectures, achieved through integrated processing and alloy design, provide a viable pathway for developing next-generation Mg alloys with improved and tunable mechanical performance.

## 1. Introduction

Owing to their exceptionally low density and high specific strength, magnesium (Mg) alloys have garnered considerable attention for deployment in weight-critical applications, including the transportation, aerospace, and energy sectors [1,2]. Beyond structural applications, Mg alloys have also emerged as promising candidates for biodegradable biomedical implants, attributable to their favorable corrosion characteristics and excellent biocompatibility [3,4,5,6]. Notwithstanding these advantages, their broader utilization remains constrained by inherently limited ductility and poor formability at ambient temperatures. These deficiencies are intrinsically linked to the hexagonal close-packed (HCP) crystallographic structure, which offers a paucity of independent slip systems and consequently engenders pronounced plastic anisotropy [7,8].

Sustained efforts have been directed toward ameliorating these limitations through alloying additions and microstructural refinement. In this context, nanostructuring paradigms have emerged as a particularly efficacious strategy [9]. The deliberate incorporation and meticulous manipulation of nanoscale defects have demonstrated substantial efficacy in simultaneously augmenting strength and ductility [10,11,12]. In the present review, the term “nanodefects” refers to nanoscale structural features and crystal imperfections that strongly influence the deformation behavior and mechanical performance of materials. These nanodefects include dislocations, stacking faults, twin boundaries, grain boundaries, solute clusters, nanograins, nanoprecipitates, nanotwins, and nano-stacking faults. Such nanodefects exert a profound influence on deformation behavior by impeding or facilitating dislocation glide, promoting the activation of non-basal slip systems, and enabling complementary mechanisms such as deformation twinning and dynamic recrystallization. A rigorous understanding of the synergistic interplay between these defect architectures and the underlying deformation mechanisms is therefore indispensable for the rational design of next-generation Mg alloys with superior mechanical performance. Despite significant advances in both experimental interrogation and computational modeling, a cohesive and critical synthesis delineating the role of diverse nanodefects in governing strengthening and deformation phenomena remains elusive [13,14,15,16].

In this review, we critically appraise the current state of knowledge pertaining to nanodefect-mediated strengthening in Mg alloys. Particular emphasis is placed on establishing robust correlations between specific defect configurations and their resultant mechanical responses across multiple length scales and temperature regimes. Furthermore, emerging defect engineering strategies and their prospective implications for the design of high-performance Mg alloys are systematically discussed.

## 2. Nanograins and Nanoprecipitates: Formation and Effects

The mechanical behavior of Mg alloys is strongly controlled by their microstructural state, especially by features introduced at the nanoscale. Owing to the limited number of independent slip systems in the HCP crystal structure of Mg, together with strong texture development and the frequent activation of deformation twinning, conventional coarse-grained Mg alloys often exhibit low room-temperature ductility, restricted formability, and anisotropic mechanical responses. Accordingly, the introduction of nanoscale microstructural heterogeneities such as high dislocation densities, nanotwins, stacking faults, shear bands, and nanocrystalline structures has emerged as an effective route to improve the strength–ductility balance and to tailor plastic deformation mechanisms. These features can enhance work hardening, delay strain localization, promote additional deformation modes, and in some cases activate grain-boundary-assisted mechanisms that are not prominent in coarse-grained materials.

**Figure 1:**
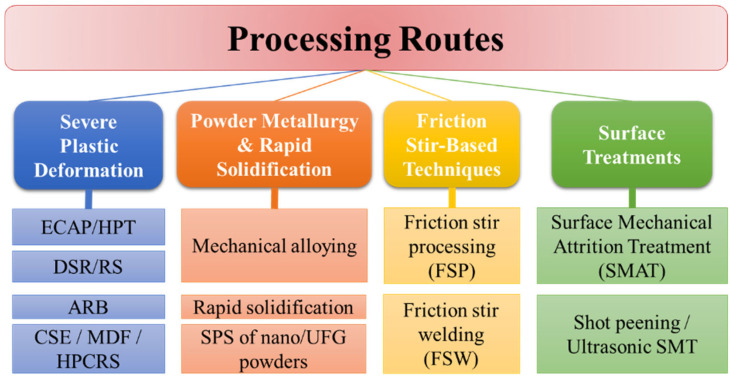
Classification of the processing routes to produce ultrafine structures in Mg alloys.

A variety of processing routes have been developed to generate such nanoscale features in Mg alloys (Figure 1). These may be broadly classified into severe plastic deformation (SPD) methods, powder metallurgy and rapid-solidification routes, friction-stir-based processing, and surface modification techniques. Although each route operates through different thermo-mechanical or kinetic pathways, they share the common objective of refining the microstructure, increasing the density of interfaces and lattice defects, and thereby improving the mechanical performance of the alloy. In addition, other repeated thermo-mechanical treatments, such as cyclic deformation, multidirectional processing, accumulative strain path changes, and repeated heat-treatment/deformation cycles, can also promote the accumulation of nanoscale defects and the formation of ultrafine recrystallized grains in Mg alloys. Among the processing variables, deformation temperature is particularly important because it strongly influences the balance between dislocation slip, twinning, recovery, recrystallization, and grain refinement. In this context, cryogenic deformation conditions are especially noteworthy, as they can suppress dynamic recovery, increase defect storage, and favour the development of high defect densities, localized twin structures, and refined recrystallized submicron grains.

### 2.1. Nanograin Structures by SPD Techniques

Unlike conventional thermo-mechanical methods, SPD allows for extensive microstructural modification by introducing enormous strains into a material, typically without the material’s overall dimensions changing drastically. Ultrafine or even nanocrystalline grains can be produced using these methods [17]. Beyond grain refinement, SPD has also demonstrated the ability to reduce porosity commonly retained from earlier fabrication methods, such as powder metallurgy. The SPD process makes it possible to create ultrafine microstructures with high-angle grain boundaries, which improve mechanical properties. SPD has demonstrated remarkable efficacy in modifying crystallographic texture and significantly enhancing the ductility of magnesium alloys. The most widely adopted SPD techniques for magnesium (Mg) alloys include equal channel angular pressing (ECAP), differential speed rolling (DSR), high-pressure torsion (HPT), rotary swaging (RS), and accumulative roll bonding (ARB). Beyond these established processing routes, several emerging SPD approaches, including continuous shear extrusion (CSE), multi-directional forging (MDF), and high-pressure compressive reverse shearing (HPCRS), have also attracted increasing attention.

**Figure 2:**
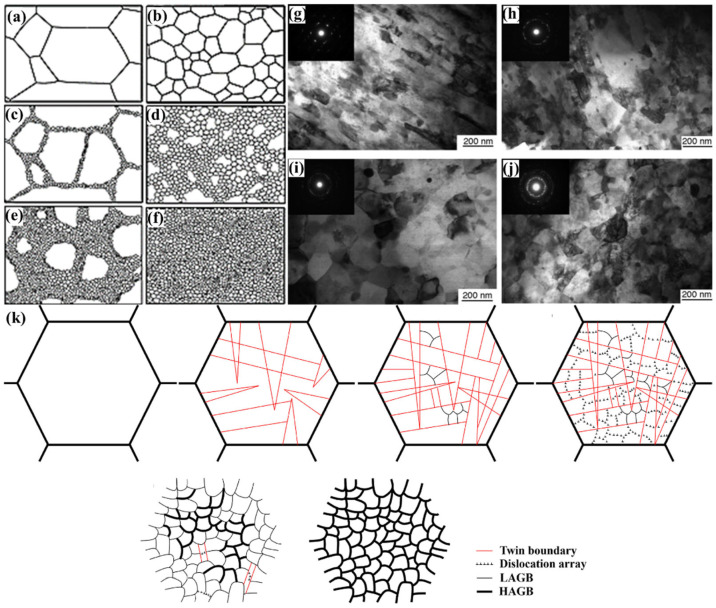
Effect of SPD processing on the grain refinement (**a**–**f**) A model for the grain refinement of magnesium alloys processed by ECAP, where (**a**,**b**) shows the initial condition, (**c**,**d**) the structure after one ECAP pass and (**e**,**f**) shows the structure after multiple passes [24]. (**g**–**j**) Images by STEM combined with the diffraction patterns for the AZ80 alloy after processing by HPT for 1/4 turn, 1 turn, 5 turns and 10 turns, respectively [27]. (**k**) Schematic representation of the nanograins formation through DRX process of the AZ31B Mg alloy during Cryogenic Rotary swaging SPD technique [31].

ECAP is widely regarded as a highly effective SPD technique due to its capability to produce ultrafine-grained microstructures. It offers considerable flexibility in processing scale, making it suitable for both laboratory studies and large-scale production. In ECAP, a billet is pressed through a die consisting of two channels with identical cross-sections that intersect at an angle, thereby imposing intense simple shear deformation. This process enables severe grain refinement and dislocation accumulation without dimensional change [18]. ECAP processing can reduce the grain size to the nanocrystalline regime, thereby significantly increasing the volume fraction of grain boundaries. This enlarged grain boundary network provides rapid diffusion pathways for hydrogen, thereby enhancing hydrogen absorption and desorption kinetics [19,20,21,22,23]. The post-ECAP microstructural evolution is strongly governed by processing parameters, particularly deformation temperature, die geometry, and the number of passes. Careful optimization of temperature and pass number is essential to regulate the extent of dynamic recovery (DRV), thereby controlling the resulting dislocation density. The processing temperature must be sufficiently elevated to suppress cracking in Mg alloys—attributable to their limited number of active slip systems at low temperatures—while simultaneously promoting the activation of non-basal slip modes and maintaining controlled grain growth. Moreover, the initial grain size plays a critical role in determining the strain accumulation required; finer or coarser starting microstructures influence the number of ECAP passes necessary to achieve the formation of an ultrafine-grained (UFG) structure. For instance, the schematic presented in Figure 2a–f, adapted from the model proposed by Figueiredo et al., illustrates the evolution of microstructure in Mg alloys during ECAP as a function of the initial grain size relative to a critical grain size [24]. As shown in Figure 2a, the alloy initially exhibits a coarse-grained microstructure where the average grain size is significantly larger than the critical grain size. Following a single ECAP pass (Figure 2c), the microstructure evolves into a heterogeneous bi-modal or multi-modal grain size distribution, characterized by the coexistence of retained coarse-grained cores and newly formed ultrafine grains. Owing to the excessively large initial grain size, the volume fraction of the coarse-grain cores remains considerable. Even after multiple passes (Figure 2e), this heterogeneity is largely preserved, indicating that markedly coarse initial structures favor the persistence of multi-modal grain distributions. A comparable, albeit less pronounced, scenario is depicted in Figure 2b, where the initial grain size remains larger than the critical size but is relatively finer than that in Figure 2a. After one ECAP pass (Figure 2d), a bi-modal grain structure is again observed; however, the fraction of newly refined grains is significantly increased, and the dominance of coarse grain regions is reduced. With continued straining through subsequent passes (Figure 2f), progressive grain subdivision and refinement lead to the development of a more homogeneous ultrafine-grained microstructure. These observations underscore the critical role of the initial grain size in governing the kinetics of microstructural homogenization during ECAP processing. When the initial grain size exceeds the critical threshold, ECAP initially produces heterogeneous grain structures. However, the extent and persistence of this heterogeneity strongly depend on the initial grain size relative to the critical size, with finer initial grains facilitating faster evolution toward a homogeneous ultrafine-grained microstructure.

On the other hand, HPT is a technique widely used to achieve extreme grain refinement in metals, often producing nanograined structures. In this process, a disk-shaped specimen is compressed between two anvils under a high hydrostatic force while being simultaneously subjected to torsional strain [25]. In the high-pressure torsion (HPT) process, grain refinement typically initiates at the disk periphery, where the shear strain is highest, and then propagates inward with increasing number of turns. This refinement is driven by intense shear deformation that first leads to a high density of dislocations and the formation of subgrains, often separated by low-angle boundaries, which gradually evolve into high-angle grain boundaries via continuous dynamic recrystallization-type mechanisms. Concurrently, shear banding concentrates deformation into localized regions, while deformation twinning subdivides parent grains and further enhances fragmentation, especially at intermediate strains; with continued torsional straining, twins may diminish as a more equiaxed, ultrafine-grained microstructure fills the entire disk [26,27,28]. For example, Alsubaie et al. investigated the evolution of microstructure in AZ80 Mg alloy processed by HPT through STEM analysis, as shown in Figure 2g–j [27]. Their observations, supported by corresponding diffraction patterns, revealed a clear transition in structural features with increasing torsional strain. In the early stages of deformation (e.g., after 1/4 and 1 turn), the microstructure was dominated by elongated grains, reflecting the initial response of the material to severe shear deformation. However, with continued processing to 5 and 10 turns, the microstructure transformed significantly into a more homogeneous arrangement of equiaxed ultrafine grains with an average size of approximately 200 nm. This refinement process was further validated through selected area electron diffraction (SAED) patterns, which showed the emergence of well-defined ring structures after higher numbers of turns. These rings are indicative of ultrafine grains separated by high-angle grain boundaries, confirming the development of a stable, refined microstructure at advanced stages of HPT. In parallel, hardness is initially heterogeneous due to non-uniform strain, but becomes increasingly uniform with higher HPT turns, achieving homogeneity after ~5 turns. Mg alloys subjected to HPT generally show poor ductility, which is mainly attributed to restricted dynamic recovery and the buildup of a high dislocation density, particularly when deformation is carried out at low temperatures. To address this limitation, post-processing heat treatment is typically introduced to facilitate static recovery, enabling partial annihilation and rearrangement of dislocations. This approach improves ductility while maintaining most of the strength achieved through severe plastic deformation [29,30].

In addition to conventional SPD techniques that primarily manipulate stress–strain conditions, cryo-deformation has emerged as an effective approach to intensify microstructural refinement [32,33]. The effectiveness of cryo-deformation lies in the strong suppression of dynamic recovery, which leads to the accumulation of significantly higher stored energy during deformation, thereby promoting enhanced grain subdivision compared to room-temperature processing [34,35]. Initially introduced in rolling, this approach has since been integrated with various SPD techniques such as multidirectional forging, equal channel angular pressing, hydrostatic extrusion, and high-pressure torsion to further refine metallic microstructures. Among these, rotary swaging (RS) represents a particularly advantageous method, especially for materials with limited workability, due to the imposition of high triaxial compressive stresses that reduce the likelihood of cracking. As an incremental forming process, RS employs radially oscillating dies to progressively reduce the cross-section of cylindrical components, enabling continuous plastic deformation and grain refinement. Chen et al. explained that grain refinement during cryogenic rotary swaging begins with extensive activation and intersection of deformation twins, which subdivide coarse grains into fine lamellae. With increasing strain, additional twins and dislocation interactions further fragment these regions into nanoscale subgrains. Due to suppressed dynamic recovery at low temperatures, high stored energy promotes dynamic recrystallization, ultimately producing equiaxed nanograins (Figure 2k). These nanograins significantly enhanced strength and hardness through grain boundary strengthening while also reducing yield asymmetry.

Overall, despite variations in processing routes and conditions, grain refinement in SPD techniques follows a common mechanism involving the progressive accumulation of dislocations, formation of subgrains, and their gradual transformation into high-angle grain boundaries, ultimately leading to ultrafine or nanocrystalline structures.

### 2.2. Grain Refinement by FSP and SMAT Techniques

**Figure 3:**
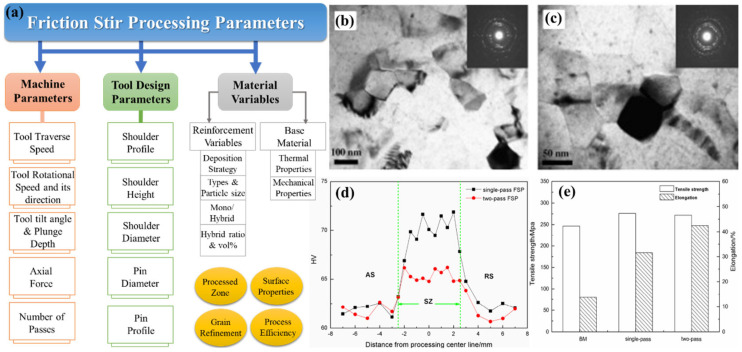
(**a**) Taxonomy of FSP parameters; (**b**,**c**) TEM micrographs of the two-pass FSP AZ31 specimens in the as-processed condition, together with the selected area diffraction patterns [44]. (**d**) Microhardness distribution of the two kinds of FSP specimens and (**e**) tensile strength and elongation of ZK60 Mg alloy produced by FSP [45].

Friction stir processing (FSP) is a solid-state technique in which process parameter selection is strongly dictated by the thermo-mechanical response of the material. Materials with high melting points or high thermal conductivity require increased heat input to ensure adequate plasticization and defect-free processing, whereas intrinsic properties such as yield strength, hardness, and ductility govern the extent of plastic flow during deformation [36,37,38]. The backing plate plays a critical role in maintaining thermal stability by acting as a heat sink while also providing mechanical constraint [39]. The detailed parameter classification for FSP is provided in Figure 3a. Additionally, FSP is recognized as an effective grain refinement technique, as it imposes severe plastic deformation under comparatively high strain rates, often exceeding those achieved in conventional SPD methods [40,41,42]. The intense plastic flow during FSP not only refines grains but also introduces a high density of dislocations and subgrain structures [43]. Additionally, the stirring action can break up second-phase particles and distribute them uniformly, contributing to dispersion strengthening. As discussed earlier, the introduction of cryogenic conditions during SPD significantly enhances grain refinement; a similar effect can be achieved in friction stir processing (FSP) through the use of liquid nitrogen as an external cooling medium. The rapid heat extraction suppresses dynamic recovery and promotes the accumulation of high defect density, thereby facilitating the formation of nanostructured grains.

For example, Chang et al. reported the development of a nanograined structure in AZ31 Mg alloy (Figure 3b,c). This refined microstructure resulted in a significant improvement in hardness, reaching ~1.5 GPa, which is nearly three times higher than that of the base material [44]. In another study by Lin et al., microstructure refinement and its effect on the mechanical properties of ZK60 Mg alloy were systematically investigated [45]. They reported that the stir zone (SZ) exhibits higher hardness than the base material due to pronounced grain refinement (Figure 3d). Whereas the elongation increases markedly due to enhanced microstructural homogeneity and phase dissolution (Figure 3e).

**Figure 4:**
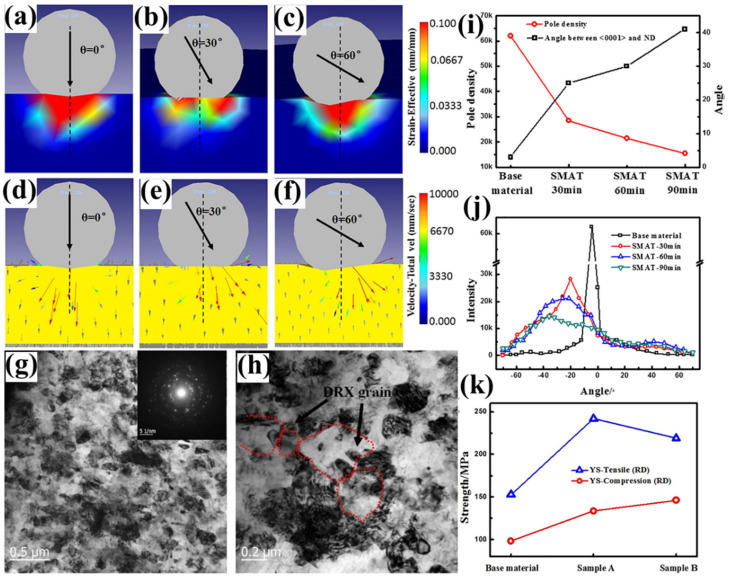
(**a**–**f**) Schematic shows how the strain and material flow during the impact process with steel balls from different directions with an angle of (**a**,**d**) 0°, (**b**,**e**) 30° and (**c**,**f**) 60° between ND and impact direction. Bright-field TEM images showing (**g**) the microstructure of the fine grain area with the corresponding SAED patterns at the top right corner and (**h**) the DRX grains along the grain boundary of the 60 min SMATed sample. (**i**) Effect of SMAT process conditions on the maximum pole density distribution and the angle between <0001> poles and ND and (**j**) the texture intensity distribution along different poles (pseudo rocking curve). (**k**) The yield strength distribution during tensile and compression tests [50].

Nanocrystalline (NC) materials, characterized by a high-volume fraction of grain boundaries, exhibit enhanced atomic diffusion compared to their coarse-grained [46] counterparts due to rapid grain boundary diffusion [47,48]. Surface mechanical attrition treatment (SMAT) has been demonstrated as an effective technique to produce such NC grains in the top surface layer of bulk materials [49], enabling the introduction of refined structures with a high density of defects. This technique relies on repeated high-energy impacts to induce severe plastic deformation at the surface. Peng et al. used hot-rolled AZ31 Mg alloy plates with thicknesses of 2.2 mm and 5 mm. As shown in Figure 4a–f, both strain distribution and material flow depend strongly on the impact direction of the steel balls. Impact along the normal direction (ND) produces a symmetric, ellipsoidal strain field with material flow aligned to ND, whereas deviations of 30° and 60° lead to asymmetric strain and deflected, more scattered material flow, reflecting changes in the stress state. These heterogeneous deformation conditions promote non-uniform strain accumulation, resulting in the formation of a gradient nanostructure, where the grain size decreases progressively from the bulk to the surface. The surface layers typically exhibit nanocrystalline grains, high dislocation densities, and residual compressive stresses. Figure 4g,h further confirms this, showing TEM observations of the surface layer after 60 min of SMAT, where a nanostructured layer with an average grain size of ~200 nm is formed, significantly refined from ~50 µm in the base material. This region also contains a high density of defects, along with evidence of ongoing dynamic recrystallization (DRX), as indicated by newly formed grains near grain boundaries.

Moreover, SMAT leads to a pronounced weakening of the basal texture, which evolves systematically with increasing treatment duration (Figure 4i,j). This is evidenced by three key features. First, the (0002) poles progressively tilt from the normal direction (ND) toward the RD-TD plane, with the inclination angle increasing from ~3° in the base material to ~41° after 90 min of SMAT. Second, the maximum pole density decreases significantly with processing time, reaching only ~25% of the base material value after 90 min.

Third, the (0002) pole contours become increasingly dispersed, as reflected by the broadening of intensity profiles from a sharp peak in the base material to a wider distribution after prolonged SMAT. The yield strength increment is found to be markedly higher under tensile loading than under compression (as shown in Figure 4k). This is attributed to the dominance of dislocation slip during tension, where the high density of SMAT-induced defects significantly impedes dislocation motion. In contrast, deformation under compression is primarily governed by {10-12} extension twinning, which is relatively insensitive to the hardened surface layer, resulting in a comparatively smaller increase in yield strength.

Conceptually, both FSP and SMAT processing techniques also fall under the category of SPD techniques, in which ultrafine- to nanocrystalline grains are produced through intense, localized shear deformation. These processes generate a very high dislocation density in the processed zone (surface layer or stirred zone), leading first to the formation of subgrains separated by low-angle boundaries, which progressively evolve into high-angle grain boundaries via continuous dynamic recrystallization-type mechanisms. Concurrently, shear banding concentrates deformation into narrow regions, while deformation twinning subdivides parent grains and accelerates microstructural fragmentation, particularly at intermediate strains; with continued straining, twinning activity is gradually suppressed and replaced by an equiaxed, nanocrystalline structure. However, the optimum choice of the technique depends on the material, geometry, desired deformation volume, and targeted microstructure. Bulk methods such as HPT or ECAP like SPD techniques provide very high, relatively homogeneous equivalent strain and are therefore especially effective for producing submicron to nanocrystalline structures throughout the entire sample volume, while localized techniques such as SMAT and FSP are more effective when the goal is to engineer only the surface or near-surface region without altering the bulk properties. The existence of multiple SPD routes thus reflects the need to balance grain-refinement capability with practical constraints such as sample size, loading configuration, temperature control, and industrial feasibility, allowing researchers and engineers to select the method that best matches the specific application requirements.

### 2.3. Formation of Nanoprecipitates and Their Effect on the Mechanical Behavior of Mg Alloys

**Figure 5:**
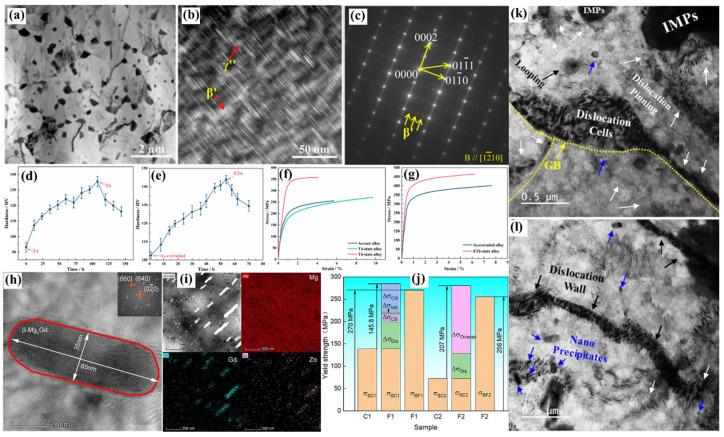
Bright-field TEM image of the Mg-Gd-Dy-Ag-Zn-Zr alloy in (**a**) as extruded form and (**b**) high-angle annular dark field (HAADF)-STEM image of the alloys after T6 treatment with (**c**) its corresponding SAED pattern. Age hardening curves of (**d**) T4 and (**e**) as-extruded alloys under 200 °C; (**f**,**g**) tensile stress–strain curves of Mg-Gd-Dy-Ag-Zn-Zr alloys with different states [52]. (**h**–**j**) Effect of multiple MDF passes on the microstructure, precipitation and strengthening of the Mg-5Li-1Zn-0.5Ag-0.5Zr-xGd (x = 2.4%, 6%) alloys [53]. (**k**,**l**) Effect of cryogenic deformation on the nanoprecipitate formation and their interactions with dislocation structures in AZX311 Mg alloy [56].

Powder metallurgy and rapid solidification techniques represent another important class of methods for introducing nanodefects. In powder metallurgy, mechanical alloying is often used to produce nanocrystalline powders through repeated cold welding, fracturing, and re-welding of particles in a high-energy ball mill. This process generates a high density of defects, including dislocations and grain boundaries, while also enabling the incorporation of alloying elements and reinforcements at the nanoscale. The consolidated powders, often processed using spark plasma sintering (SPS), retain much of their refined microstructure due to the rapid heating and short sintering times, which suppress grain growth. Rapid solidification techniques, such as melt spinning, involve extremely high cooling rates that prevent the formation of equilibrium phases and lead to supersaturated solid solutions and ultrafine grains. Rapid solidification can produce supersaturated solid solutions and ultrafine grains, and it may also quench into defects such as vacancies and dislocation loops that later act as nucleation sites for nanoprecipitates during aging or subsequent processing. For instance, Tang et al. showed that body-centred cubic Mg-Li-Al alloys can undergo exceptional immediate strengthening after solution treatment and water quenching, which they attributed to the precipitation of semi-coherent D0_3_-type Mg_3_Al nanoprecipitates during rapid cooling from the solution-treated state [51]. In their work, the rapid quenching process traps a supersaturated matrix and promotes the formation of a high density of nanoscale Mg_3_Al precipitates that initially strengthen the alloy; however, with prolonged low-temperature ageing, these nanoprecipitates gradually coarsen and lose coherency, leading to a subsequent decrease in strength. During the deformation process, the aging response of Mg alloys can be significantly enhanced by introducing a high density of potential nucleation sites, thereby accelerating the precipitation kinetics and increasing the population of nanoprecipitates. Concurrently, hot deformation promotes grain refinement, and because Mg alloys exhibit a relatively high Hall–Petch slope, the reduction in grain size substantially contributes to their overall strength. Accordingly, combining deformation-induced grain refinement with an increased number of nanoprecipitates offers an effective strategy for designing high-performance Mg alloys. Figure 5a shows TEM micrographs of the extruded Mg-10Gd-4Dy-1.5Ag-1Zn-0.5Zr alloy, where hot extrusion induces submicron-sized dynamic precipitates along the grain boundaries of DRXed grains. Figure 5b,c illustrate the microstructural evolution after ageing, as revealed by TEM analysis at the peak-aged (T6) condition. In the aged matrix, two distinct types of nanoprecipitates with mutually perpendicular orientations are observed. SAED patterns taken along the [11,12,13,14,15,16,17,18,19,20] zone axis display strong streaks parallel to the α direction, indicating the formation of γ″-type nanoprecipitates. Additional diffraction spots at the 1/4{01-10}_α_, 2/4{01-10}_α_, and 3/4{01-10}_α_ positions (marked by yellow arrows in Figure 5c) are also detected, which are attributed to β′-type nanoprecipitates with an elliptical morphology. It was further investigated by Xie et al. that the β′ nanoprecipitates have an average length of about 20.94 nm and a width of 10.81 nm, whereas the γ″ nanoprecipitates exhibit an average length of approximately 35.64 nm and a thickness of around 2 nm. The corresponding volume fractions of β′ and γ″ nanoprecipitates are estimated to be about 8% and 13%, respectively [52]. This microstructural variation is reflected in the age-hardening behavior shown in Figure 5d,e. The T4 alloy requires more than 108 h to reach peak hardness (~136 HV) from an initial value of ~93 HV, whereas the as-extruded (ET6) alloy achieves its peak hardness of ~144 HV in only ~54 h, indicating that the pre-deformed state accelerates precipitation strengthening. The shorter peak-ageing time and higher ultimate hardness of the ET6 condition are consistent with the finer and more uniformly distributed nanoprecipitates (β′ and γ″) observed in the aged matrix, which enhance dislocation pinning and overall resistance to plastic flow. The improved precipitation state and refined microstructure also translate into superior tensile performance, as illustrated in Figure 5g,h. Both peak-aged alloys exhibit markedly increased strength relative to their respective as-received states, yet the ET6 alloy outperforms the conventional T6 counterpart. The yield strength and ultimate tensile strength of the T6 alloy reach ~312 MPa and ~356 MPa, respectively, classifying it as a typical high-strength Mg casting alloy. In contrast, the ET6 condition achieves ~396 MPa and ~451 MPa, clearly placing it in the category of high-strength Mg wrought alloys. This performance gap can be attributed to the combined effect of grain refinement, grain-boundary precipitates introduced during extrusion, and the higher volume fraction and finer dispersion of β′ and γ″ nanoprecipitates in the deformed and aged matrix.

Precipitates act as obstacles to dislocation glide during plastic deformation, thereby enhancing the mechanical properties of the alloy through precipitation strengthening. When the precipitates are small and coherent with the matrix, strengthening is typically governed by the Orowan bowing mechanism, in which dislocations loop around the particles rather than cutting through them. In the regime where coherent shearing still occurs, the contribution to strength can be rationalized via three main mechanisms. The first contribution arises from coherency strengthening, which originates from the elastic interaction between the strain fields of coherently misfit precipitates and dislocations [53]. The second contribution stems from modulus strengthening, which results from differences in the shear moduli of the precipitate and the matrix [54]. The third contribution is associated with ordered strengthening, in which matrix dislocations shear ordered precipitates and generate an antiphase boundary (APB) on the slip plane [55]. The magnitude of ordered strengthening is primarily determined by the antiphase boundary energy associated with the interaction between dislocations and ordered precipitates. In this context, Qian et al. characterized the APB structure formed by dislocation-coherent precipitate interactions using high-resolution TEM and illustrated the underlying formation mechanism, providing direct experimental support for the role of ordered nanoprecipitates and their APB-mediated strengthening in Mg-based alloys (Figure 5h–j). In their work, the Mg-5Li-1Zn-0.5Ag-0.5Zr-xGd (x = 2.4, 6) alloys were subjected to multi-directional forging (MDF) at 350 °C. The alloys with 2.4 wt.% Gd and 6 wt.% Gd were designated as C1 and C2, respectively, in the pre-forged condition, and as F1 and F2 after MDF processing. It was found that the increased Gd content gives rise to β-Mg_5_Gd nanoprecipitates with dimensions of approximately 85 nm × 35 nm (Figure 5h). EDS mapping in Figure 5i confirms that these particles are enriched in Mg, Zn, and Gd, which supports their assignment to the β-Mg_5_Gd phase; quantitative analysis estimates the corresponding precipitate volume fraction at about 4.0%. During MDF, the F2 alloy exhibits enhanced β-phase precipitation, and the strengthening mechanism shifts with precipitate size. Larger β-phase particles effectively impede twin-boundary migration, leading to bent twin boundaries. As further illustrated in Figure 5j, the F2 alloy exhibits a superior yield strength improvement of 207 MPa compared to the C2 state, a significant gain directly attributed to the synergistic interaction between dislocations and β-Mg_5_Gd nanoprecipitates. This enhancement is primarily governed by the dislocation bypass (Orowan) mechanism, as the larger, incoherent β-phase particles effectively impede dislocation motion and pin twin boundaries. Quantitative analysis confirms that this strengthening increment is a consequence of the high density and volume fraction of these nanoprecipitates, which serve as potent barriers to deformation. This strengthening mechanism was also observed by Tariq et al. in AZX311 Mg alloys deformed at cryogenic temperatures, where TEM analysis revealed nanoprecipitate acting as potent barriers to dislocation motion (Figure 5k,l) [56].

The dislocations form pile-ups around these precipitates, as indicated by the white arrows, which demonstrates that nanoprecipitates effectively pin dislocations and obstruct their glide. When a moving dislocation encounters such a non-shearable precipitate, it initially undergoes Orowan bowing; subsequently, it forms a loop around the particle, a process known as Orowan looping, highlighted by the black arrow in Figure 5k. This bypass mechanism generates back-stresses that impede further dislocation propagation, directly contributing to the increase in the strength of the alloy [57].

**Figure 6:**
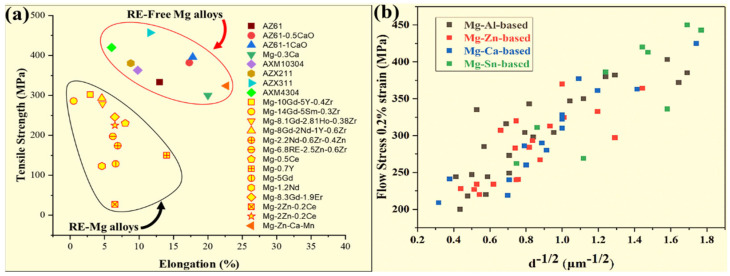
(**a**) A scatter plot comparing the tensile strength and elongation of various Mg alloys containing RE and non-RE elements [35,67,68,69,70,71,72,73,74,75,76,77,78,79,80]. (**b**) Yield stress vs. d^−1/2^ plot of different alloy systems [66].

A persistent challenge in the development of low-cost wrought Mg alloys is their limited absolute strength. While precipitation hardening via high-density second phases is a standard strengthening strategy, the age-hardening response in Mg alloys remains modest compared to Al alloys. Specifically, precipitation strengthening contributions are typically restricted to ~40 MPa in Mg-Al/Sn-based alloys and about 70 MPa in M-Zn-Zr systems [58,59,60]. Even in highly alloyed Mg-RE-Zn systems, the strengthening increment is generally limited to ~170 MPa [61]. Although the solubility of individual RE elements in Mg is relatively limited, the combined addition of multiple RE elements enables the formation of a supersaturated solid solution. This subsequently decomposes through a well-defined precipitation sequence involving GP zones, followed by β″, β′ (bcc), and ultimately the equilibrium β (fcc) phase [62], thereby providing enhanced resistance to dislocation motion. These precipitates play a key role in strengthening by impeding dislocation motion. For example, Mg alloys containing 8 wt.% Gd and 3 wt.% Nd achieve a UTS of 271 MPa [63]. Even higher strength, up to 355 MPa, has been reported in Mg–8Gd–1Dy–0.4Zr alloys, attributed to the presence of finely dispersed Mg_15_RE_2_ and Mg_5_RE precipitates [64]. The strengthening effect of these precipitates is primarily associated with their ability to hinder dislocation slip, particularly on basal planes. Moreover, the coexistence of prismatic β-type and basal γ-type precipitates can lead to a mutually orthogonal arrangement, forming an effective barrier network against dislocation motion and thereby significantly enhancing the strength [65]. An additional advantage of RE-containing precipitates is their relatively high thermal stability, as their melting temperatures exceed that of the Mg matrix. This ensures sustained strengthening performance even at elevated temperatures [16]. Despite these advantages, the widespread industrial adoption of Mg-RE alloys is constrained by high material costs, processing complexity, and recyclability issues. Consequently, significant efforts have been directed toward developing RE-free Mg alloys with comparable mechanical performance. Several alternative alloying elements have demonstrated promising strengthening effects. The alloying addition of Mn traces to Mg-Al-Ca alloys simultaneously improves strength and ductility, increasing the yield strength (YS) from 366 MPa to ~400 MPa and elongation from ~2.8% to ~4% [66]. A broader comparison of strength and ductility between RE-containing and RE-free Mg alloys is presented in Figure 6a, while Figure 6b illustrates the Hall–Petch relationship for various RE-free systems, including Mg-Al, Mg-Zn, Mg-Ca, and Mg-Sn alloys [67]. These results indicate that ultra-high strengths exceeding 450 MPa can be achieved in RE-free Mg alloys primarily through effective grain refinement.

## 3. Planar Defect Engineering: Nanotwins and Nano-Stacking Faults

In HCP Mg alloys, where plastic deformation is constrained by a limited number of active slip systems, twin boundaries (TBs) act as key microstructural features governing mechanical behaviour [81,82,83,84]. These TB interfaces effectively regulate dislocation motion, thereby contributing to strength and ductility [33,85]. Unlike conventional high-angle grain boundaries (HAGBs), TBs possess enhanced thermal and mechanical stability, particularly at nanoscales [86]. Therefore, exploiting coherent twin interfaces has gained increasing attention as an effective strengthening strategy, comparable to established approaches such as grain refinement and precipitation hardening [87].

In Mg alloys, the most frequently observed deformation twin modes are {10-12} tension twins (TTWs) and {10-11} contraction twins (CTWs). TTWs are primarily activated to accommodate tensile strain along the c-axis, whereas CTWs accommodate compressive strain in the same crystallographic direction. The interface of TTWs is relatively unstable and tends to migrate forward during deformation, often engulfing entire grains, which is why TTW spacing commonly remains in the range of several to tens of micrometres. In contrast, CTWs typically exhibit a finer scale and can nucleate secondary tension twins within their interior, leading to the formation of {10-11}–{10-12} double twins (DTWs). Due to the inherent instability of TTW interfaces, these secondary tension twins may grow until they fully occupy the CTW domain, thereby restricting further reduction in twin spacing. As a result, stress tends to concentrate at twin-twin junction interfaces, which can contribute to premature failure. For a long time, it was believed that achieving high strength–ductility synergy via twin-based mechanisms in Mg alloys was not feasible. The main reason is that stress concentrations at DTW boundaries or twin tips are poorly relaxed by non-basal slip, owing to the high critical resolved shear stress ratio between prismatic/non-basal slip and basal slip. Consequently, voids and microcracks tend to initiate near DTWs. This implies that the deformation twins are not the primary cause of failure; rather, it is the limited capacity for plastic relaxation through additional slip systems that governs premature failure. For example, the study by Tariq et al. on AZX311 Mg alloy shows that twin boundaries can contribute positively to mechanical performance when supported by sufficient non-basal slip [8]. Their work reveals orientation-independent yield strength and superior tensile properties at 90° to the rolling direction, attributed to enhanced pyramidal <c+a> slip and multiple twin variants, including {10-12} TTWs and {10-11}–{10-12} DTWs. In this case, TBs form stably and continuously during deformation, sustaining strain hardening at higher strains, while increased non-basal slip alleviates stress concentrations at twin interfaces, leading to improved strength–ductility synergy.

**Figure 7:**
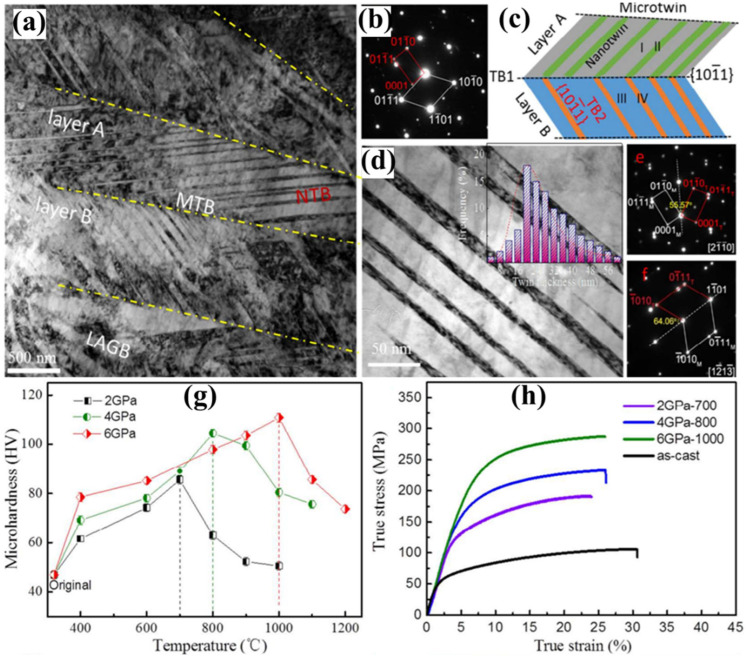
Microstructural characteristics of the 6 GPa-1000 Mg-8Li alloy. (**a**) Bright field TEM image of the 6GPa-1000 sample. (**b**) SAED patterns of LAGB area. (**c**) Schematic of hierarchical {10-11}-{10-11} double contraction NTWs. (**d**) Enlargement of the {1010} NTWs, the inset corresponds to the thickness distribution of the {1010} NTWs. (**e**) SAED patterns of NTW interfaces along [2-1-10] direction. (**f**) SAED patterns of NTW interfaces along [1-21-3] direction. (**g**) Microhardness and (**h**) tensile curves of Mg-8Li alloys prepared under different conditions [88].

In the broader context of Nano twinning-based strengthening in HCP Mg alloys, coherent nanotwins have been increasingly recognized as effective microstructural features that combine high strength with reasonable ductility by providing dense, semi-coherent interfaces that both block dislocations and accommodate slip transfer. In this framework, the work of Fu et al. on the Mg-8Li alloy employs ultrahigh-pressure treatment (2–6 GPa) combined with controlled high-temperature annealing (200–1200 °C) to induce hierarchical nanotwins (NTWs) within the as-cast material. The bright-field TEM image (Figure 7a) reveals two types of microstructures: a fine band-shaped matrix of grains containing low-angle grain boundaries having no twins (Figure 7b), representing ~35 vol.% of the microstructure, and a hierarchical double-twin structure occupying the remaining ~65 vol.%. Within the twin-dominated regions, macro-twins (MTWs) with thicknesses of ~1–2 µm host internal NTWs averaging ~20 nm in thickness, which account for ~10 vol.% of the MTW domain (Figure 7d). SAED pattern (Figure 7e,f) at the NTW boundaries shows full overlap of the (10-11)_I_ and (10-11)_II_ diffraction spots along the <2-1-10> zone axis, indicating an orientation relationship [2-1-10]_I_ ∥[2-1-10]_II_ with (10-11)_I_ ∥(10-11)_II_. The SAED pattern shows that the layer I rotates ~56.41° to overlap with the layer II about <2-1-10>, which is consistent with the conventional {10-11} CTW, and the same orientation relation is found between lamellae III and IV in a second twin variant (layer B). On this basis, the twinning elements identified by Fu et al. are (10-11) and [10-1-2], together with their reciprocal counterparts, which collectively establish that the interior {10-11}-NTW structure nested within the {10-11}-MTW corresponds to a novel {10-11}–{10-11} double compression twin (DCTW). This hierarchically organized nanotwin architecture is directly linked to the exceptional mechanical response observed in the same alloy (Figure 7g,h). A peak-like dependence of microhardness on treatment temperature at fixed pressure is reported, with the as-received alloy hardness (~50 HV) increasing by over 200% after processing at 6 GPa and 1000 °C. The peak hardness reaches ~108 HV at 6 GPa, which is ~1.37 times higher than that at 2 GPa and represents the highest value yet reported for Mg-Li-based systems, outperforming those achieved by conventional alloying, aging, or severe deformation routes. The increase in treatment pressure also elevates the effective melting point and shifts the temperature of peak hardness to higher values, indicating that the nanotwinned state is thermodynamically stabilized under these extreme conditions. In tensile testing (Figure 7h), the 6 GPa-1000 sample exhibits a dramatic enhancement in both yield strength and ultimate tensile strength, while retaining a high elongation of ~23.6%, the best strength–ductility synergy. This behavior can be attributed to the high density of coherent {10-11} DCTWs and nanotwin interfaces, which efficiently impede dislocation motion, promote more uniform strain partitioning, and stabilize the microstructure against detwinning and damage incubation.

**Figure 8:**
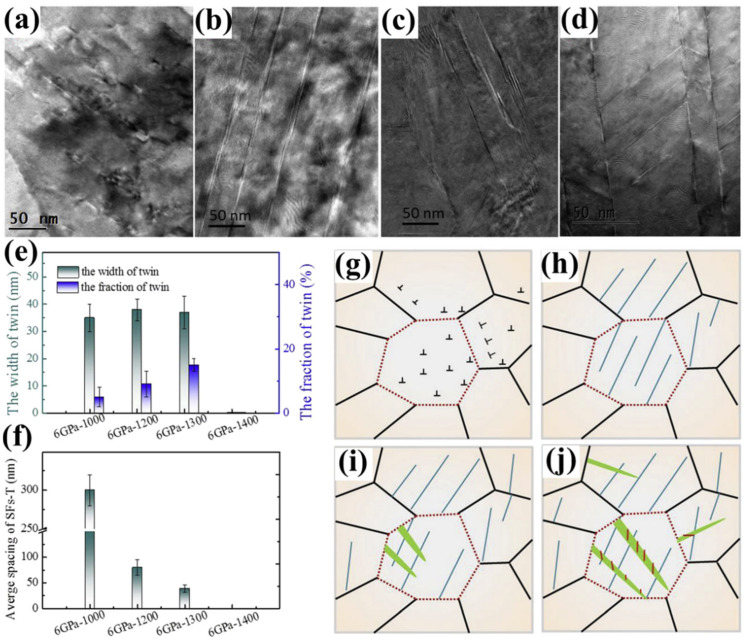
Structural evolution. (**a**) TEM image of the as-rolled Mg-13Li sample. (**b**) TEM image of the CR-UPed 6 GPa-200 Mg-13Li alloy. (**c**) TEM image of the CR-UPed 6 GPa-1000 Mg-13Li alloy. Some short and fine contraction TBs form during the UPed treatment. Few SFs are detected in the interior of CTWs. (**d**) TEM image of the CR-UPed 6 GPa-1200 Mg-13Li alloy. Both the length of TB and the density of SFs increase. (**e**) The effect of temperature on both the width and the fraction of twins. (**f**) The variation in the average spacing of SFs-T is dependent on temperature. (**g**–**j**) Schematics of structural evolution during the CR-UPed treatment [91].

Stacking faults (SFs) are usually planar defects at the nanoscale in crystalline metals that arise primarily from the motion of partial dislocations. From an energy perspective, the formation of partial dislocations is favoured over full dislocations because they encounter a lower energy barrier for glide on closely packed crystallographic planes. As a result, an SF develops behind a leading partial dislocation, while a trailing partial subsequently forms to partially or fully restore the regular stacking sequence. The equilibrium width of the SF is determined by the balance between the stacking fault energy and the elastic energy of the two partial dislocations. In this way, SFs act as effective barriers to further dislocation motion, producing pronounced pinning effects that can significantly influence the flow stress and work-hardening behaviour. The Basal-plane stacking faults (SFs) are commonly observed in Mg-based systems and are expected to play roles similar to twin boundaries in blocking <c+a> dislocation slip [89]. Theoretically, each SF corresponds to a mismatched atomic layer, such that the volume fraction of SFs can reach at least twice that of twin lamellae. This high interfacial density makes the design of interactive contraction twin-stacking fault (CTWSF) architectures particularly attractive, as it not only suppresses detwinning but also enables a substantially increased fraction of coherent interfaces [90], offering a promising route to simultaneously enhance strength and ductility in HCP Mg alloys. For instance, Peng et al. investigated the contraction nanotwins-stacking faults strengthening mechanism in Mg-13Li alloy induced by cryo-rolling followed by the ultrahigh-pressure (CR-UPed) technique. The loading pressure was fixed to 6 GPa with the holding temperature varied from 400 to 1400 °C. To elucidate the influence of temperature, ex situ TEM was employed to examine the microstructure evolution of CR-UPed Mg-13Li alloy treated at 6 GPa across a range of temperatures. In contrast to the as-rolled sample, which is dominated by a high density of dislocations introduced by cryo-rolling pre-treatment (Figure 8a), the CR-UPed 6 GPa-400 Mg-13Li alloy exhibits a pronounced population of nanoscale stacking faults (SF-M) arising from dislocation rearrangement, with an average SF-M spacing of 35 ± 6 nm (Figure 8b). When the ultrahigh-pressure treatment temperature is increased to 1100 °C (Figure 8c), nano-sized {10-11} CTWs begin to appear, while the density of SF-M is markedly reduced and SF-T at the twin interfaces becomes nearly undetectable. The presence of intense strain fields at the tops of the CTWs suggests that nanoscale CTW formation is closely linked to localized micro-strain processes under high pressure. Upon further raising the UPed temperature to 1200 °C (Figure 8d), the CTW width remains relatively stable, whereas the fraction of stacking faults increases significantly, indicating that the CTWSF structure emerges as a synergistic product of both temperature and pressure. Statistical analysis (Figure 8e,f) reveal that the twin width is largely maintained in the range 1000–1300 °C, while the twin volume fraction increases and the average SF-T spacing decreases, highlighting the gradual transition from a stacking-fault-dominated to a twin-plus-fault architecture. The overall structure evolution under different UPed conditions is schematically summarized in Figure 8g–j.

These observations also clarify the origin of the enhanced mechanical properties. The initial cryo-rolling stage contributes mainly through a high density of trapped dislocations. In the early CR-UPed regime, the formation of SF-M by dislocation arrangement becomes the dominant strengthening mechanism, echoing effects previously documented in Mg-RE alloys [91]. At higher temperatures, the emergence of nanoscale CTWs leads to a rapid rise in strength, with the subsequent peak hardness associated with the combined contributions of CTWs and SF-T. The sharp decline in mechanical performance at 1400 °C is primarily attributed to the collapse of the CTWSF architecture through re-solidification-related processes (as discussed above), which effectively removes the stabilizing nanoscale twins and stacking faults.

**Figure 9:**
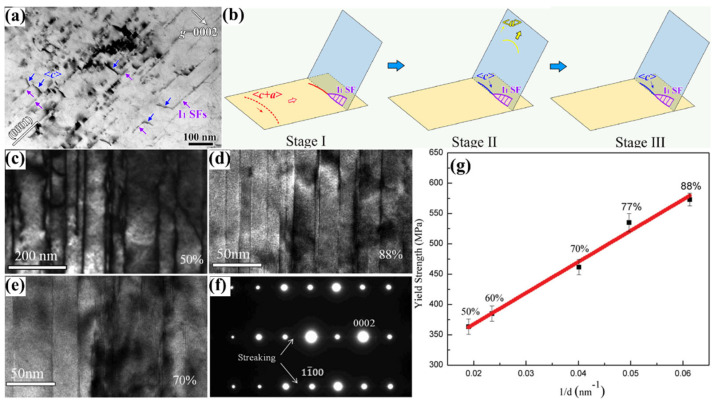
(**a**) Formation mechanism of connected <c> dislocations and stacking faults in pure Mg showed by a bright field TEM image, viewing from the direction of g = 0002, (**b**) schematic diagram of the process of dislocation dissociation [93]. TEM images of Mg alloy samples with various rolling thickness reductions (**c**) 50%, d = 55 nm, (**d**) 70%, d = 25 nm and (**e**) 88%, d = 16 nm. (**f**) The SAD pattern of the 70% rolled sample in which the streaking verifies the basal plane SFs. (**g**) Yield strength vs. the reciprocal of mean spacing between SFs of rolled samples with different thickness reduction. The number by each data point indicates the thickness reduction by hot rolling [92].

In medium- and fine-grained samples, a frequently observed configuration is the attachment of <c> dislocation tips to I_1_ stacking faults (Figure 9a), giving the appearance of a <c> dislocation blocked by an I_1_ fault. Both <c> dislocations and I_1_ stacking faults are known to be sessile in HCP alloys, making it more plausible that such structures arise from growth or dissociation processes rather than simple pinning. A schematic of a proposed formation mechanism is shown in Figure 9b. A <c+a> dislocation gliding on a pyramidal plane is inherently unstable and tends to dissociate, forming an I_1_ fault ribbon whose width is inversely proportional to the stacking fault energy. In one region, the <c+a> dislocation partially dissociates into leading and trailing partials, temporarily pinning the dislocation (Stage I). Elsewhere, the same <c+a> dislocation dissociates into a sessile <c> dislocation and a glissile <a> dislocation (Stage II). As SFs exert little resistance to perfect <a> dislocations, the <a> segment slips away, leaving the <c> dislocation connected to the I_1_ fault (Stage III).

Moreover, the spacing between SFs plays a critical role in determining their strengthening effect. The introduction of a high density of nanoscale-spaced SFs provides a large number of coherent barriers that effectively block and pin dislocations, while simultaneously promoting retained work hardening and enhanced ductility. Figure 9c–f shows that the formation of basal plane SFs becomes the primary crystalline defect after 50% of the thickness reduction by hot rolling in Mg-8.5Gd-2.3Y-1.8Ag-0.4Zr alloy [92]. The average spacing between adjacent SFs decreases progressively with increasing rolling reduction. At a thickness reduction of approximately 88%, nano-spaced SFs with a mean spacing of ∼16 nm are observed to form within the majority of the coarse grains (~13 μm), reflecting the development of a high-density SF network. This behaviour is attributed to the relatively low stacking fault energy (SFE) of the present Mg-Gd-Y-based alloy, which facilitates the formation of large populations of planar faults during plastic deformation.

The relationship between SF spacing and yield strength was further analyzed by using a Hall–Petch-type relationship (Figure 9g). The large slope of the linear correlation between strength and 1/d, where d is the average SF spacing, indicates that stacking faults are highly effective in strengthening the material. Specifically, introducing nano-spaced SFs with a mean spacing of ~16 nm increased the yield strength of the Mg alloy by about 70%, from 340 MPa to 575 MPa, underscoring the marked strengthening contribution of a high-density SF network. This strength enhancement is achieved without significant loss of ductility, which highlights the promise of stacking-fault engineering as a strategy for simultaneously improving both strength and ductility in Mg alloys.

Nanograins, nanoprecipitates, nanotwins, and stacking faults all strengthen Mg alloys, but they do so in different ways and with different trade-offs in strength and ductility. Nanograins mainly increase strength by blocking dislocation motion at grain boundaries and by changing the texture, although too much grain refinement can reduce strain hardening and make the structure less stable at high temperatures. Nanoprecipitates strengthen the alloy by hindering dislocations through coherency, cutting, or bypassing mechanisms, and they can be very thermally stable, especially in Mg-RE alloys; however, they may lose effectiveness as they coarsen or become over-aged, and high alloying costs can also be a drawback. Nanotwins are attractive because their coherent interfaces can block dislocations while still allowing some plastic deformation, which helps balance strength and ductility, but stress can concentrate at twin intersections or double twins if non-basal slip is limited. Stacking faults also act as planar barriers to dislocation motion and can support work hardening, but their formation and stability depend strongly on stacking-fault energy, composition, and processing. In the end, no single defect type is best in every case; the strongest Mg alloys usually come from combining multiple nanoscale defects in a well-designed microstructure.

## 4. Synergistic Interactions Among Nanodefects and Their Effects on Mechanical Properties

The formation and stabilization of nanograins and nanoscale defects in Mg alloys are governed by the interplay between thermodynamic driving forces and kinetic constraints. From a precipitation thermodynamics perspective, the nucleation of nanoprecipitates is governed by the balance between bulk chemical free-energy reduction (ΔG_v_), interfacial energy (γ), and strain energy associated with lattice misfit. In Mg-RE systems, precipitation sequences such as SSSS → β″ → β′ → β1 → β are strongly dictated by supersaturation and diffusion thermodynamics [94]. Metastable β′ nanoprecipitates in Mg-Gd-Y or Mg-Nd alloys are particularly effective strengthening phases because their coherent or semi-coherent interfaces reduce nucleation barriers while simultaneously impeding dislocation glide through shearing or Orowan looping mechanisms. Alloy chemistry also plays a crucial thermodynamic role by altering stacking fault energy (SFE), solute segregation tendencies, and defect stability. Rare-earth additions have been reported to reduce basal stacking fault energy and modify generalized planar fault energy landscapes, thereby facilitating non-basal <c+a> slip, nanotwin formation, and stacking-fault stabilization. Kinetically, nanodefect generation is highly sensitive to temperature, strain rate, diffusion mobility, and deformation pathway. Low-temperature and high-strain-rate processing suppress diffusion-controlled recovery and grain boundary migration, leading to elevated dislocation densities and enhanced defect retention. Cryogenic deformation of Mg alloys, for instance, markedly suppresses dynamic recovery while promoting deformation twinning and dislocation storage due to reduced cross-slip and climb kinetics. Conversely, elevated-temperature processing enhances atomic mobility, enabling DRX, solute redistribution, precipitate growth, and grain coarsening. The kinetics of recrystallization in Mg alloys are additionally influenced by the inherently anisotropic HCP crystal structure, where restricted basal slip activity necessitates the activation of deformation twinning, pyramidal <c+a> slip, and grain-boundary-mediated plasticity. In nanostructured Mg alloys, twin boundaries, stacking faults, and high-density interfaces frequently act as both defect sinks and preferential diffusion pathways, thereby altering recovery and precipitation kinetics.

The mechanical response of Mg alloys is not governed by isolated defect species; rather, it emerges from the intricate and often non-linear interactions among multiple nanoscopic defect populations [95]. Dislocations, twins, grain boundaries, SFs, and solute clusters coexist and dynamically interact under applied stress, giving rise to synergistic phenomena that transcend the contributions of individual defects. Such interactions are pivotal in reconciling the long-standing strength–ductility trade-off in HCP systems [96]. One of the most prominent examples of such synergy is the interplay between dislocations and deformation twins. Twin boundaries not only act as effective barriers to dislocation motion, thereby contributing to strain hardening, but also serve as sources and sinks for dislocations [97]. The impingement of dislocations on twin interfaces often leads to their absorption, transmission, or re-emission, depending on the local crystallographic orientation and stress state. This dynamic interaction facilitates the activation of non-basal slip systems, thereby enhancing plastic accommodation and mitigating anisotropic deformation. Concurrently, the refinement of twin lamellae introduces additional interfaces that contribute to Hall–Petch-type strengthening. Grain boundaries, particularly in ultrafine-grained and nanocrystalline Mg alloys, further amplify these synergistic effects. They interact with both dislocations and twins, leading to complex mechanisms such as twin nucleation at grain boundaries and grain boundary-mediated plasticity [94]. The presence of solute clusters or segregated alloying elements at these interfaces can stabilize grain boundary structures, impede boundary migration, and modulate twin propagation. This phenomenon not only enhances strength through boundary pinning but also contributes to improved thermal stability and creep resistance [98,99]. SFs and solute atoms introduce additional layers of complexity by altering the intrinsic SFE of the matrix. A reduced SFE promotes partial dislocation activity and facilitates the nucleation of deformation twins, whereas solute-dislocation interactions give rise to solid solution strengthening and dynamic strain aging effects [100]. The concomitant presence of these defects often results in a hierarchical strengthening mechanism, wherein dislocation glide is successively hindered by solute atmospheres, stacking faults, twin boundaries, and grain boundaries.

Importantly, the collective operation of these nanodefects can engender strain hardening mechanisms that are both sustained and adaptive. For instance, the sequential activation of basal slip, prismatic or pyramidal slip, and twinning mediated by evolving defect structures enables Mg alloys to accommodate larger plastic strains without premature failure. This hierarchical and interactive deformation behavior is particularly pronounced under extreme conditions, such as cryogenic or elevated temperatures, where defect mobility and interaction kinetics are markedly altered.

The influence of deformation temperature on nanodefect generation and deformation behavior in Mg alloys is commonly interpreted using temperature-dependent constitutive and kinetic relations rather than a single unified model. The empirical form of the model is given by [101,102];

$$Z=\dot{\varepsilon }exp(\frac{QR}{T})$$

where $\dot{\varepsilon }$ is strain rate, *Q* is activation energy, *R* is the gas constant, and *T* is absolute temperature. High *Z* conditions (low temperature/high strain rate) generally promote dislocation accumulation, twinning, and grain refinement through suppressed dynamic recovery.

$$\dot{\varepsilon }=A[sinh\left(\alpha \sigma \right){]}^{n}\;exp(\frac{-Q}{RT})$$

This relation links flow stress (*σ*) to thermally activated dislocation motion, dynamic recovery, and recrystallization kinetics during deformation. The flow stress can be written as a function of *Z* by the following relationship;

$$\sigma =\frac{1}{\alpha }ln\{(\frac{Z}{A}{)}^{n}+[(\frac{Z}{A}{)}^{\frac{2}{n}}+1{\}}^{\frac{1}{2}}\}$$

where *D* is diffusivity. Increasing temperature enhances atomic mobility, thereby accelerating recovery, recrystallization, grain boundary migration, and precipitate growth/coarsening.

Deformation temperature also influences slip and twinning activity through changes in the CRSS required for activating different deformation modes. Increasing temperature generally lowers the CRSS for non-basal slip in Mg alloys, facilitating pyramidal <c+a> slip and altering the slip–twinning balance.

Despite these advances, a comprehensive mechanistic framework that quantitatively captures the cooperative behavior of multiple defect species remains underdeveloped. Future efforts should therefore focus on integrating high-resolution experimental techniques with multiscale modeling approaches to elucidate the spatiotemporal evolution of interacting defects. Such insights will be instrumental in advancing defect engineering strategies aimed at designing Mg alloys with an optimal balance of strength, ductility, and environmental resilience.

## 5. Challenges and Future Perspectives

Despite substantial advances in understanding nanodefect-mediated deformation mechanisms in Mg alloys, several critical challenges persist, hindering the development of a unified, predictive framework for property optimization. Foremost among these is the intrinsic complexity associated with the concurrent evolution of multiple interacting defect populations under service conditions. The spatiotemporal coupling between dislocations, twins, grain boundaries, stacking faults, and solute clusters introduces a high degree of non-linearity in deformation behavior, rendering mechanistic decoupling and quantitative modeling particularly challenging.

Another key limitation is the difficulty of experimentally resolving defect interactions at the requisite spatial and temporal scales. Although advanced characterization techniques such as aberration-corrected transmission electron microscopy (AC-TEM), three-dimensional atom probe tomography (3D-APT), and in situ electron microscopy have significantly enhanced our understanding, capturing real-time defect evolution during active deformation remains non-trivial. Consequently, many proposed mechanisms are still inferred from post-mortem microstructural observations rather than direct dynamic evidence.

From a theoretical standpoint, existing computational models often operate across limited length and time scales, restricting their ability to fully capture hierarchical defect interactions. Bridging atomistic simulations with continuum-scale constitutive models remains an unresolved challenge, particularly for HCP metals, where anisotropic slip and twinning mechanisms must be considered simultaneously. The absence of robust, multiscale predictive frameworks continues to impede the rational design of Mg alloys with tailored mechanical responses.

Looking ahead, future research should focus on quantitatively understanding the influence of defect size, density, spacing, and interface characteristics on the mechanical behavior and thermal stability of advanced materials. In particular, systematic investigations on nanotwin spacing, dislocation density, nanoprecipitate distribution, grain boundary character, and nano-stacking fault interactions are required to establish clear structure–property relationships. The integration of in situ characterization techniques with physics-based multiscale modeling can provide deeper insight into the dynamic evolution of these nanodefects during deformation and thermal exposure. Furthermore, machine learning-assisted microstructure–property correlation models trained on experimentally validated datasets may accelerate the optimization of defect hierarchies for achieving a superior balance of strength, ductility, creep resistance, and structural stability.

## 6. Conclusions

This review demonstrates that nanodefect engineering provides a powerful framework for overcoming the intrinsic strength–ductility limitations of Mg alloys. Among the different defect types, nanograins remain one of the most effective strengthening approaches due to strong Hall–Petch hardening and texture modification, although excessive refinement may reduce thermal stability and strain hardening capacity. Nanoprecipitates, particularly fine and coherent precipitates, offer efficient strengthening through precipitate shearing, coherency/modulus/order strengthening, and Orowan bypassing, while also providing improved thermal stability in suitable alloy systems. Planar defects, including nanotwins and nano-stacking faults, are efficient because their coherent interfaces can simultaneously impede dislocation motion and facilitate plastic accommodation, thereby offering strong potential for improved strength–ductility synergy. A key take-home message is that the mechanical response of Mg alloys is governed not by isolated defects but by synergistic defect architectures, such as grain refinement coupled with precipitation strengthening, nanotwin-assisted deformation combined with non-basal slip activation, and stacking-fault/interface engineering. However, several challenges still constrain practical alloy design, including controlling defect stability during thermal exposure, managing competing strengthening–ductility trade-offs, reducing dependence on costly RE alloying strategies, and achieving scalable, reproducible processing routes. Future advances will therefore depend on integrated control of processing, alloy chemistry, and hierarchical defect design, supported by deeper mechanistic understanding and predictive design approaches.

## Data Availability

No new data were created or analyzed in this study. Data sharing is not applicable to this article.

## Abbreviations

The following abbreviations are used in this manuscript:

HCP	Hexagonal Close-Packed
SPD	Severe Plastic Deformation
ECAP	Equal Channel Angular Pressing
DSR	Differential Speed Rolling
HPT	High Pressure Torsion
RS	Rotary Swaging
CSE	Continuous Shear Extrusion
MDF	Multi-directional forging
HPCRS	High-Pressure Compressive Reverse Shearing
UFG	Ultrafine Grain
SFE	Stacking Fault Energy
CTWs	Compression Twins
LAGB	Low-Angle Grain Boundary
HAGB	High-Angle Grain Boundary
APB	Anti-Phase Boundary
SAED	Selected Area Electron Diffraction
DCTWs	Double-Compression Twins
TTWs	Tension Twins
NTWs	Nano Twins
MTWs	Macro Twins
TEM	Transmission Electron Microscope
